# Effects of Whole Milk Supplementation on Gut Microbiota and Cardiometabolic Biomarkers in Subjects with and without Lactose Malabsorption

**DOI:** 10.3390/nu10101403

**Published:** 2018-10-02

**Authors:** Xiaoqin Li, Jiawei Yin, Yalun Zhu, Xiaoqian Wang, Xiaoli Hu, Wei Bao, Yue Huang, Liangkai Chen, Sijing Chen, Wei Yang, Zhilei Shan, Liegang Liu

**Affiliations:** 1 Department of Nutrition and Food Hygiene, Hubei Key Laboratory of Food Nutrition and Safety, School of Public Health, Tongji Medical College, Huazhong University of Science and Technology, Wuhan 430030, China; immary@163.com (X.L.); m201575208@hust.edu.cn (J.Y.); 15926237664@163.com (Y.Z.); M201675194@hust.edu.cn (X.W.); M201675193@hust.edu.cn (X.H.); hyhy199310@163.com (Y.H.); D201678154@hust.edu.cn (L.C.); sijingchen19@sina.com (S.C.); yw8278@hotmail.com (W.Y.); zshan@hsph.harvard.edu (Z.S.); 2 Ministry of Education Key Lab of Environment and Health, School of Public Health, Tongji Medical College, Huazhong University of Science and Technology, Wuhan 430030, China; 3 Department of Epidemiology, College of Public Health, University of Iowa, Iowa City, IA 52242, USA; wei-bao@uiowa.edu; 4 Departments of Nutrition, Harvard T.H. Chan School of Public Health, Boston, MA 02115, USA

**Keywords:** lactose malabsorption, milk, gut microbiota, *Bifidobacterium*, short-chain fatty acids

## Abstract

The aim of this study was to compare the impact of whole milk supplementation on gut microbiota and cardiometabolic biomarkers between lactose malabsorbers (LM) and absorbers (LA). We performed a pair-wise intervention study of 31 LM and 31 LA, 1:1 matched by age, sex, body mass index, and daily dairy intake. Subjects were required to add 250 mL/day whole milk for four weeks in their routine diet. At the beginning and the end of the intervention period, we collected data on gut microbiota and cardiometabolic biomarkers. Whole milk supplementation significantly increased Actinobacteria (*P* < 0.01), *Bifidobacterium* (*P* < 0.01), *Anaerostipe* (*P* < 0.01), and *Blautia* (*P* = 0.04), and decreased *Megamonas* (*P* = 0.04) in LM, but not LA. Microbial richness and diversity were not affected. The fecal levels of short-chain fatty acids (SCFAs) remained stable throughout the study. Body fat mass (*P* < 0.01) and body fat percentage (*P* < 0.01) reduced in both groups, but the changes did not differ between groups. No significant differences in other cardiometabolic markers were found between LM and LA. When compared with LA, whole milk supplementation could alter the intestinal microbiota composition in LM, without significant changes in fecal SCFAs and cardiometabolic biomarkers.

## 1. Introduction

Milk has been commonly regarded as an essential component of a balanced diet, since it is rich in high-quality protein, calcium, as well as plenty of health-promoting bioactive components, such as whey peptides, conjugated linoleum acid, oligosaccharides, and immunoglobulin [1]. Previous prospective studies suggested that milk consumption was significantly associated with a lower risk of obesity [2], hypertension [3], type 2 diabetes [4], and cardiovascular disease [5]. In humans, the ability to digest lactose, which is the main carbohydrate present in milk, depends on the levels of the enzyme lactase-phlorizin hydrolase (LPH) in the small intestine, which decline rapidly after weaning in a majority of humans. Lactose malabsorption occurs when there are not enough LPH to hydrolyze ingested lactose in them. Under this condition, undigested lactose is generally fermented by colonic microbiota into short-chain fatty acids (SCFAs) and gases (CO_2_, H_2_, and CH_4_), which could lead to abdominal bloating, abdominal pain, diarrhea, and other uncomfortable symptoms [6]. Although previous evidence suggested that even the severe lactose malabsorbers (LM) could tolerate the lactose in 240 mL milk per day [7], lactose malabsorption was proposed to account for low dairy intake in many populations [8,9]. For instance, the prevalence rate of lactose malabsorption is up to 80% in the Chinese population [10]. According to the results of the 2010–2013 China Nutrition and Health Surveillance, the average dairy consumption was only 24.7 g/day in Chinese adults [11], which is much lower than the recommendation of 300 g/day [12].

In lactose malabsorbers, the undigested lactose and products derived from its fermentation in the colon could be energy sources of some gut bacterial species and alter the gut microbiota composition, so the impact of milk intake on the gut microbiota composition may be different in subjects with and without lactose malabsorption. Lactose was demonstrated to have the capacity to stimulate the growth of putatively beneficial genera, such as *Bifidobacterium* in LM, but not in lactose absorbers (LA) [13]. However, intervention studies are sparse to examine the board-spectrum response of microbiota community to lactose/milk stimulation, including both expected cross-feeding interaction and changes in the gut environment [14]. Additionally, accumulating evidence has suggested that alternations in gut microbiota composition could affect host lipid and glucose metabolism via SCFAs production through carbohydrate fermentation, and then contribute to metabolic disease [15,16]. Consequently, milk intake may lead to changes in the cardiometabolic profiles through the alternations in gut microbiota in LM. However, to our best knowledge, no previous study has comprehensively investigated the effects of milk intervention on the gut microbiota composition and cardiometabolic health, according to lactose absorption status.

Therefore, in the current study, we aimed to investigate the potentially differential effects of whole milk supplementation on the gut microbiome and cardiometabolic biomarkers in LM and LA.

## 2. Materials and Methods

Prior to initiating the study, the study design and consent form were reviewed and approved by the Ethics Committee of the School of Public Health, Tongji Medical College, Huazhong University of Science and Technology (approval No. 12012015). The study was registered at clinicaltrials.gov (NCT02798718).

### 2.1. Study Participants

This study was a 1:1 pair-wise matching intervention study in lactose absorbers and malabsorbers. At the beginning, 233 participants were voluntarily recruited from Tongji Medical College of Huazhong University of Science and Technology. The inclusion criteria included healthy volunteers aged ≥18 years, Han population, and average dairy intake less than one serving during the past year. We then excluded the participants with a history of acute or chronic gastrointestinal disorders, any known metabolic disease (diagnosed diabetes, hypertension, or cardiovascular disease), consumption of antibiotics or probiotics within the preceding month, use of medications or dietary supplements that could influence glucose or lipids metabolism, pregnancy or lactation, excessive alcohol consumption, significant body weight variation in the past three months, or a known allergy to milk.

Then, 227 eligible participants underwent a hydrogen breath test after lactose administration to examine the presence of lactose malabsorption. Written informed consent was obtained from all the participants before participating. This test was performed with a hydrogen breath test analyzer (Shenzhen Zhonghe Headway Bio-Sci & Tech Co., Ltd., Shenzhen, China). Briefly, after a standardized low-carbohydrate dinner and a 12-h fast, the end-expiratory breath H_2_ was determined before and at 30-min intervals after a 25-g lactose load for the ensuing 3 h. Eating, smoking, and exercising were not allowed throughout the test. A rise of 20 ppm in breath H_2_ over basal values was considered as an indication of lactose malabsorption [17]. Finally, 31 LA and 31 LM were recruited by 1:1 pair-wise matching with age (±3 years), sex, body mass index (BMI) (±10%), and the average dairy intake (<125 g/day, 125–250 g/day) for the intervention study. The details of participant recruitment are shown in Supplementary Figure S1.

### 2.2. Milk Intervention and Dietary Intake Assessment

In one-week run-in phase, all of the participants were required to maintain their initial diet. In the following four-week intervention, they were further instructed to add extra one box of 250 mL whole milk (Mengniu Dairy (Group) Co., Ltd., Hohhot, China) in their routine diet per day. All of the subjects were instructed to maintain their usual physical activity throughout the entire study. The participants were asked to return the empty milk package to assure compliance.

To quantify macronutrient composition, individuals were instructed to complete a three-day food record (including two weekdays and one weekend day) at the end of run-in and intervention periods. Before start of the study, the participants were instructed by investigators how to weigh and record their food intake. The dietary records were checked, in the case of missing data, and analyzed by the investigators. Energy and nutrient intake were calculated according to the “Chinese Food Composition Tables” [18].

### 2.3. Cardiometabolic Biomarkers Measurement

Anthropometric and biochemical measurements were conducted at the beginning and end of the intervention period. Body composition was measured by bioelectric impedance analysis using Inbody 720 (BIOSPACE CHINA Inc., Shanghai, China). Body weight (kg) and body height (cm), were measured with standardized techniques. BMI (kg/m^2^) was calculated as body weight divided by the square of height. Waist circumference, hip circumference, and blood pressure were measured with standardized techniques.

Fasting blood samples were drawn from an antecubital vein into heparinized tubes in the morning after a 12-h fast. Plasma was separated in a 4 °C centrifuge and stored at −80 °C until analysis. Fasting plasma glucose (FPG), total cholesterol (TC), triglycerides (TG), low-density lipoprotein cholesterol (LDL-C), and high-density lipoprotein cholesterol (HDL-C) were analyzed by colorimetric enzymatic methods using commercial kits (BioSino Bio-Technology & Science Inc., Beijing, China). Fasting plasma insulin (FPI) and C-peptide were analyzed with ELISA kits (Mercodia AB, Uppsala, Sweden). C-reactive protein (CRP) was determined with ELISA kits (R&D Systems, Inc., Minneapolis, MN, USA). Malondialdehyde (MDA) was measured according to the thiobarbituric acid method with commercial kits (Jiancheng Bioengineering Institute, Nanjing, China). Intraassay variation for FPG, TC, TG, LDL-C, HDL-C, FPI, C-peptide, CRP, and MDA ranged from 0.9% to 5.5% and interassay variation ranged from 1.2% to 6.5%. Homeostasis model assessment of insulin resistance (HOMA-IR) was calculated from fasting glucose and insulin while using the following formula [19]:

HOM-IR = [FPI (mU/L) × FPG (mmol/L)] ÷ 22.5
(1)

### 2.4. Fecal Samples Collection and DNA Extraction

Participants were instructed to collect their stool using aseptic swabs and were sealed dung cup before and after the intervention study. Uncontaminated samples were collected and immediately stored in a provided cooler, then frozen at −80 °C within 30 min. DNA extraction was performed using a QIAamp Fast DNA Stool Mini Kit (Qiagen, Valencia, CA, USA). The concentration of bacterial DNA was measured using Nanodrop 2000 (Thermo Scientific, Wilmington, NC, USA).

### 2.5. 16S Ribosomal RNA Gene Sequencing

The V3-V4 region of the bacteria’s 16S ribosomal RNA (rRNA) gene was amplified with barcode-indexed primers (338F and 806R) by thermocycler Polymerase Chain Reaction (PCR system (GeneAmp 9700, ABI, Hampton, NH, USA) using FastPfu Polymerase. Amplicons were purified using AxyPrep DNA GelExtraction Kit (Axygen Biosciences, Union City, CA, USA), then quantified using QuantiFluor-ST (Promega, Madison, WI, USA). Sequencing was performed using the Illumina MiSeq platform (Illumina, San Diego, CA, USA).

### 2.6. Fecal Microbiota Analysis

The 16S rRNA sequencing data were processed using the Quantitative Insights Into Microbial Ecology platform (QIIME, Boulder, CO, USA, V.1.7.1) [20]. Raw sequencing reads were demultiplexed and filtered. Chimeras were removed and the operational taxonomic units (OTUs) were with 97% homology were generated using USEARCH [21]. The identified taxonomy was aligned with Ribosomal Database Project Classifier [22] and Greengenes reference database (V.13.8). To ensure an even sampling depth, all of the samples were rarefied to the lowest read number. Alpha and beta diversity metrics were calculated in QIIME using the rarefied OTU table. Beta diversity was estimated using weighted and unweighted UniFrac distances between samples and was visualized with principal coordinate analysis (PCoA).

### 2.7. Fecal SCFA Analysis

Fecal samples (0.5 g) were homogenized after addition of 5 mL of ultrapure water and centrifuged at 4000 rpm for 10 min at 4 °C. The supernatant fluid (600 μL) was mixed with 120 μL 25% phosphoric acid, homogenized and centrifuged at 12,000 rpm for 10 min at 4 °C. Then, the supernatant was filtered through a Millex-GS 0.22-mm syringe filter unit (Millipore, Burlington, MA, USA). The filtrate was mixed with 2-ethylbutyrate (DRE, Altshausen, Germany) as the internal standard with 1 mM. A mixed-SCFA standard solution was prepared by using analytical quality (>99% purity) reagents (Sigma-Aldrich, Bornem, Belgium). SCFAs were quantified with an Agilent 6890N gas chromatograph coupled with an Agilent 5975B mass spectrometer (Agilent Technologies Santa Clara, CA, USA). The capillary GC column was an Agilent HP-INNOWAX 30 m × 0.25 mm, 0.25 μm film thickness, with helium as the carrier gas at a constant flow rate of 2.8 mL/min. The GC oven temperature program was as follows: initial temperature 72 °C, then to 170 °C at 13 °C/min, to a final temperature 230 °C, where it was held for 5 min, at 30 °C/min. The temperatures of the injector, transfer line, and detector were set at 200 °C, 280 °C, and 220 °C, respectively. The total run time was 14.54 min. Acetate, propionate, and butyrate were quantified with appropriate calibration curves obtained using internal standard quantitation. The Varian MS workstation software (version 6.6) was used for data acquisition and processing. The interday and intraday coefficient of variances ranged from 4.6% to 8.7%.

### 2.8. Quantitative Polymerase Chain Reaction (qPCR)

PCR amplification and detection were conducted in 96-well plates with PrimeScript^TM^ RT reagent kit (TAKARA BIO INC., Dalian, China) on a DNA Engine Opticon 2 fluorescence detection system (BIO-RID, Hercules, CA, USA). Samples were run with a final volume of 25 μL, containing 0.4 μM of each primer and 2 μL of the respective template DNA. The specific primers for *Bifidobacterium* forward CTCCTGGAAACGGGTGG and reverse GGTGTTCTTCCCGATATCTACA [23]. Amplifications were done with the following temperature profiles: one cycle at 95 °C (30 s), 40 cycles of denaturation at 95 °C (5 s), 61 °C (30 s), and 72 °C (45 s), and a final one cycle of 94 °C (5 min). Quantification was done by using standard curves made from know concentrations of plasmid DNA, which was prepared according to Sabine et al. [24].

### 2.9. Statistical Analysis

All the data were expressed as the mean ± standard error of the mean (SEM). Dietary energy and nutrients intake were analyzed using Student’s paired *t*-test. For cardiometabolic biomarkers, baseline differences were evaluated using a Student’s unpaired *t*-test. Between-group differences were analyzed using a two-factor repeated measures analysis of variance (ANOVA) with time (pre, post) and group (LM, LA) as the factors. In the case of a significant time effect being shown, post-hoc analyses with Bonferroni correction were applied to identify significant within-group differences. The Kolomogorov-Smirnov test was performed to test the normality of distribution. If skewed distribution, data was logarithmically transformed. Statistics were performed with SPSS 24.0 (IBM, Brussels, Belgium).

For phylum- or genus-level microbiota groups, comparisons between two groups were evaluated with Mann-Whitney tests, within-group differences were analyzed using Wilcoxon signed-rank tests, between-group differences were evaluated using a linear mixed model with package “lme4” taking into account of repeated measurements, group, gender, and BMI. Data of qPCR were expressed as logarithmically transformed. ANOVA with Turkey post-hoc analysis was applied to compare the alpha diversity between and within groups. Beta diversity was assessed based on unweighted and weighted UniFrac distances and its significance was determined using permutation-based ANOVA with the use of the Adonis function in the “vegan” package. *P* values were corrected for multiple comparisons using the Benjamini-Hochberg procedure. Enterotyping on the basis of Partitioning Around Medoids with Jensen-Shannon divergence was conducted on a combined genus-abundance matrix of all samples [25]. Besides, the optimal number of cluster was determined by the Calinski–Harabasz Index. Statistics were performed using R version 3.3.3. A two-tailed *P* < 0.05 was considered to be statistically significant. A corrected false discovery rate (FDR) < 0.2 was considered as significant [26].

## 3. Results

A total of 62 subjects, 31 LA and 31 LM, participated in the intervention study. There were 44 men and 18 women with an average age of 24.7 ± 0.3 years and an average body mass index of 22.0 ± 0.4 kg/m^2^. No subjects dropped out of the study during the intervention period (See Supplementary Figure S1). No significant differences were found between LA and LM at the baseline (Table 1). No adverse events and gastrointestinal discomforts were reported during the entire study. Energy and macronutrient intake, as assessed by self-reported three-day food records, remained stable throughout the study, but calcium intake significantly increased in all of the participants following the whole milk intervention (See Supplementary Table S1).

### 3.1. Fecal Microbiota

**Figure 1:**
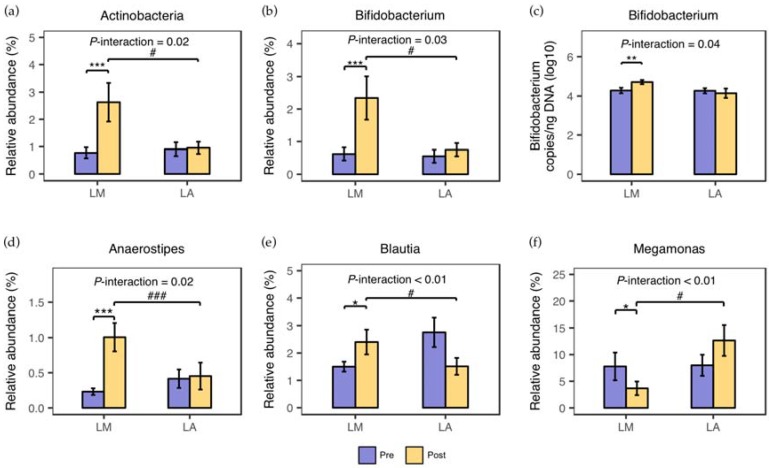
Alternations in gut microbiota composition following four-week supplementation of whole milk in lactose malabsorbers (LM) and absorbers (LA). (**a**–**f**), The relative abundance of Actinobacteria (**a**), *Bifidobacterium* assessed using Illumina 16S rRNA gene sequencing (**b**) and quantitative real-time Polymerase Chain Reaction (**c**), *Anaerostipes* (**d**), *Blautia* (**e**), and *Megamonas* (**f**) before and after 4-week supplementation of whole milk in LM (*n* = 31) and LA (*n* = 29, two participants were excluded during the gut microbiota analysis owing to antibiotics consumption during the intervention period for upper respiratory tract infection). Data are presented as Mean ± SEM (*, *P* < 0.05, **, *P* < 0.01, ***, *P* < 0.001 for within-group difference; ^#^, *P* < 0.05 and ^###^, *P* < 0.001 for between-group difference).

**Figure 2:**
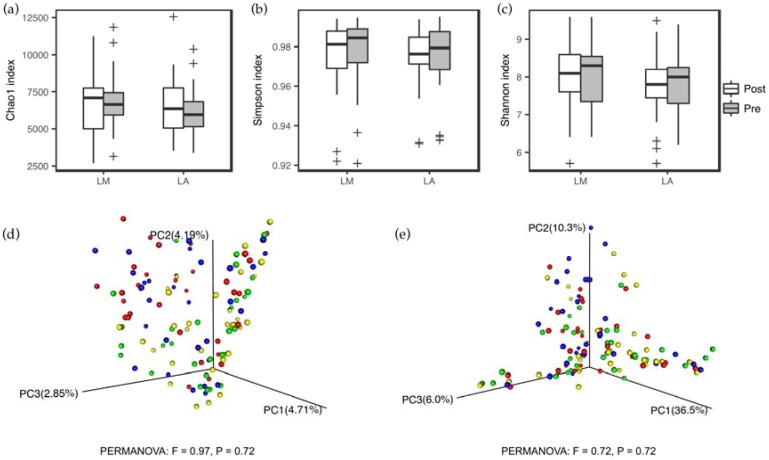
Gut microbiota diversity remained stable throughout the entire study. (**a**–**c**), α-Diversity, illustrated by Chao1 index (**a**), Simpson index (**b**), and Shannon index (**c**), of lactose malabsorbers (LM, *n* = 31) and absorbers (LA, *n* = 29). (**d**,**e**) Principal coordinate analysis based on unweighted (**d**) and weighted (**e**) UniFrac analysis of the microbiota communities in LA at baseline (red) and week 4 (blue), and LM at baseline (yellow) and week 4 (green). Box represents the interquartile range, the line inside represents the median, whiskers represent 10–90 percentiles, “+” represents outliers that are past the ends of the whiskers.

At baseline, the microbiota composition at the phylum and genus level (See Supplementary Table S2) did not differ between LM and LA. Four-week whole milk supplementation induced significant alternations in microbiota composition of LM, but not that of LA (Figure 1). At the phylum level, Actinobacteria dramatically increased in LM (1.86 ± 0.67%, *P* < 0.01, FDR < 0.01). At the genus level, *Bifidobacterium* determined by Illumina 16S rRNA gene sequencing steep increased in LM (1.72 ± 0.62%, *P* < 0.01, FDR < 0.01), which was mirrored by qPCR analysis. Besides, there were a significant increase in *Anaerostipe* (0.77 ± 0.19%, *P* < 0.01, FDR < 0.01) and *Blautia* (0.90 ± 0.48%, *P* = 0.04, FDR = 0.11), and a significant decrease in *Megamonas* (4.11 ± 2.02 %, *P* = 0.04, FDR = 0.11) in LM. Between-group analysis highlighted the significant interaction of lactose absorption status and whole milk supplementation on Actinobacteria (*P* for interaction = 0.02), *Bifidobacterium* (*P* for interaction = 0.03), *Anaerostipe* (*P* for interaction = 0.02), *Blautia* (*P* for interaction < 0.01), and *Megamonas* (*P* for interaction < 0.01). No significant variations in microbiota diversity were found within and between the groups throughout the entire study (Figure 2).

**Figure 3:**
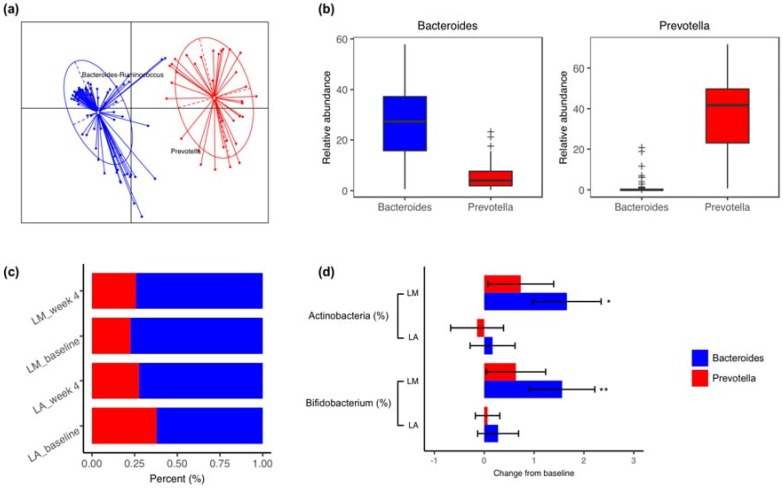
Enterotypes stratification and changes in gut microbiota and clinical variables by enterotypes. (**a**) The first two principal coordinates of Jensen-Shannon distances based on the relative abundance profiles at the genus level. (**b**) Relative abundance of bacterial taxa characteristic of each enterotype. Box represents the interquartile range, the line inside represents the median, whiskers represent 10–90 percentiles, “+” represents outliers that are past the ends of the whiskers. (**c**) Sample distribution over enterotypes before and after whole milk supplementation in lactose malabsorbers (LM) and absorbers (LA). (**d**) Comparisons of changes in the microbiota abundance in LM and LA with different enterotypes (LM with *Bacteroides* enterotype, *n* = 24; LA with *Bacteroides* enterotype, *n* = 18; LM with *Prevotella* enterotype, *n* = 7; LA with *Prevotella* enterotype, *n* = 11). Data are presented as Mean ± SEM (*, *P* < 0.05, and **, *P* < 0.01).

Fecal communities were then clustered into *Bacteroides* and *Prevotella* enterotypes (Figure 3). Although the sample distribution over enterotypes was not affected by he lactose malabsorption status (*P* = 0.43) or whole milk supplementation (*P* = 0.84), shifts in the gut microbiota composition differed between enterotypes (Figure 3). At the phylum level, Actinobacteria merely increased in LM with *Bacteroides* enterotype (1.66 ± 0.69%, *P* = 0.02, FDR = 0.02), but not in LM with *Prevotella* enterotype. Similarly, at the genus level, a similar trend was observed in *Bifidobacterium* (1.56 ± 0.65%, *P* < 0.01, FDR = 0.07).

### 3.2. Fecal Short-Chain Fatty Acids Concentrations

**Figure 4:**
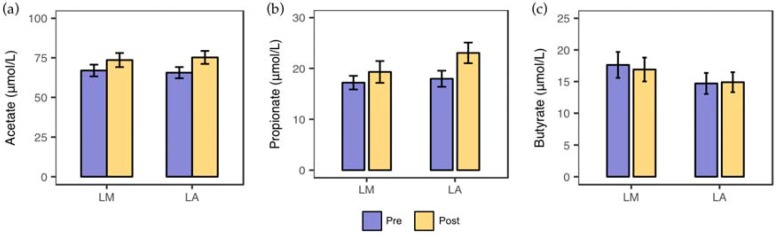
Fecal concentrations of short-chain fatty acids before and after whole milk supplementation in lactose malabsorbers (LM) and absorbers (LA). Fecal acetate (**a**), propionate (**b**), and butyrate (**c**) concentrations before and after four-week supplementation of whole milk in LM (*n* = 31) and LA (*n* = 31).

Fecal acetate, propionate, and butyrate concentration did not significantly differ within and between the groups (Figure 4).

### 3.3. Body Composition and Cardiometabolic Markers

Although body weight and BMI remained unchanged during the intervention study, body fat mass (*P* = 0.03 for LM; *P* < 0.01 for LA) and the proportion of body fat (*P* = 0.02 for LM; *P* < 0.01 for LA) significantly decreased in both groups (Table 2). Changes in body composition did not differ between LM and LA. No significant within- or between-group differences were observed in blood pressure, FPG, FPI, C-peptide, TG, TC, LDL-C, HDL-C, CRP, and MDA. Change in LDL-C was inversely associated with a change in *Blautia* abundances among LM (*P* = 0.01), but no shifts in the cardiometabolic biomarkers were associated with alternations in the relative abundance of *Bifidobacterium*, *Anaerostipes*, and *Megamonas* (See Supplementary Tables S3–S6).

## 4. Discussion

In the present study, as compared with LA, four-week 250 mL/day whole milk supplementation selectively altered gut microbiota composition in LM, especially in those with *Bacteroides* enterotype, without significant effects on the overall microbiota richness and diversity. Additionally, no significant differences were found in alternations in fecal SCFAs or cardiometabolic biomarkers between LM and LA.

We observed that four-week whole milk supplementation significantly increased the relative abundances of *Bifidobacterium* in LM, which was mainly consistent with a previous study, showing the bifidogenic effects of lactose in healthy LM in western population [13]. *Bifidobacterium* could utilize lactose as a preferable energy source rather than glucose [27], and thus the abundance of *Bifidobacterium* could increase with higher lactose intake derived from milk supplementation [28]. *Bifidobacteria* is believed to play a pivotal role in gut microbiota homeostasis and human health [29]. Several strains in *bifidobacteria* have been widely used as probiotics because of both direct and indirect influences on enhancing immunity, inhibiting the growth of pathogenic bacteria, and treating inflammatory disease [30]. In our study, the abundance of *Anaerostipes* and *Blautia* also increased with whole milk supplementation in LM. *Anaerostipes* was a butyrate-production bacteria, and the cross-feeding relationship between *Anaerostipes* and *Bifidobacterium* might account for its alteration [31]. *Blautia* was acetogenic and might benefit from the production of hydrogen, a production from lactose fermentation by *Bifidobacterium* [32]. Additionally, our results indicated that whole milk supplementation induced different alternations in microbiota composition across different enterotypes, which was in line with previous findings that individuals with distinct enterotypes differed in responses to the same diet intervention [33,34].

There were no significant changes in the fecal levels of SCFAs following four-week supplementation of whole milk, despite the significant changes in several SCFA-producing microbiota taxa. One potential explanation for this might be conversion among SCFAs through microbial cross-feeding, in particular, from acetate to butyrate [35], which was mainly metabolized in the colon to supply energy for colonocytes [36]. But, some studies suggested that fecal butyrate significantly increased following 8-week intervention of undigested carbohydrate intervention, but did not following four-week intervention [15]. Therefore, relative short intervention duration might be another reason accounting for the unchanged fecal concentrations of SCFAs in this study, and long-term studies are needed to further verify this finding in the future.

In this study, whole milk supplementation did not significantly affect the cardiometabolic biomarkers, except slight decreases of body fat mass and body fat percentage in LM and LA. Several intervention studies suggested that consumption of dairy products might have a beneficial effect on cardiometabolic factors [37,38,39,40]. A meta-analysis of randomized controlled trial elucidated that dairy products reduced body fat mass in short-term intervention studies, but not in long-term [37], which well supported the results of our study. A possible explanation might be the differential compliance with the intervention protocol between short and long trials [41].

In our study, the marked microbial changes in LM did not affect carbiometabolic biomarkers. Previous studies showed that *Bifidobacteria* improved diabetes induced by high-fat diet [29,42] but we did not observe any association between *Bifidobacterium* and carbiometabolic biomarkers. When compared the *Bifidobacterium* of our study population with that of other population, we found the baseline abundance of *Bifidobacterium* was markedly lower in our study population, but it reached slightly higher abundance after whole milk population as compared to this healthy, untreated population [26,43]. Therefore, one reason for lack of effects on the cardiometabolic biomarkers in LM may be the significant but small increase as well as inconsistent pattern of increase. Our observations indicated that moderate whole milk supplementation (250 mL/day) had no adverse cardiometabolic effect in LM, compared with LA. Accordingly, it is possibly acceptable for LM to consume moderate whole milk per day for health promotion, which would draw more attention in the countries of high prevalence of lactose malabsorption.

There are several strengths of the current study. To our best knowledge, no previous study has comprehensively compared the effects of whole milk supplementation on the gut microbiota composition and its crucial metabolites (SCFAs) and cardiometabolic biomarkers in LM and LA. We also investigated the possible different responses to whole milk in subjects with different enterotypes. Our study also has certain limitations. Firstly, we did not include a group without whole milk intervention, so the potential effects of non-treatment factors could not be excluded. However, the 1:1 pair-wise design allowed us to evaluate the different effects of whole milk supplementation between LM and LA. Secondly, we did not control the diet of the participants, but added whole milk to the initial diet. However, total energy and macronutrients intake was not different between two groups, and the effects of changes in diet could be excluded. Thirdly, four weeks may be relative short to observe changes in cardiometabolic biomarkers of healthy adults aged from 20–30 in this study, whose cardiometabolic biomarkers maintain a relatively steady state. Long-term studies or studies in the participants of different metabolic statuses are needed to replicate our findings and precisely assess the effect of milk on cardiometabolic outcomes.

## 5. Conclusions

In conclusion, when compared with LA, 250 mL/day whole milk supplementation could alter the intestinal microbiota composition in LM, without alternations in fecal SCFAs and metabolic biomarkers. It may be acceptable to include moderate milk as a part of the routine diet of LM, as a suggestion to personalize milk consumption, and thereby enhance the levels of dietary calcium intake and improve health. Further studies are warranted to confirm our findings and clarify the underlying mechanisms.

## Figures and Tables

**Table 1:** Baseline characteristics of subjects with and without lactose malabsorption ^1^.

Parameters	LM (*n* = 31)	LA (*n* = 31)	*P*
Gender (Male/Female)	22/9	22/9	1.00
Age (years)	24.7 ± 0.4	24.8 ± 0.4	0.86
Height (cm)	170.0 ± 1.4	169.6 ± 1.4	0.86
Weight (kg)	62.8 ± 2.2	64.5 ± 2.1	0.58
BMI (kg/m^2^)	21.6 ± 0.6	22.3 ± 0.6	0.42
Waist-hip ratio	0.81 ± 0.01	0.82 ± 0.01	0.57
DBP (mmHg)	75.6 ± 1.1	75.7 ± 1.7	0.98
SBP (mmHg)	115.9 ± 1.9	116.2 ± 2.4	0.92
FPG (mmol/L)	5.14 ± 0.06	5.15 ± 0.07	0.89
FPI (mU/L)	5.72 ± 0.63	5.86 ± 0.64	0.91
HOMA-IR	1.30 ± 0.15	1.36 ± 0.16	0.89
TG (mmol/L)	0.74 ± 0.07	0.86 ± 0.06	0.09
TC (mmol/L)	4.22 ± 0.15	4.19 ± 0.22	0.93
LDL-C (mmol/L)	2.52 ± 0.14	2.39 ± 0.14	0.51
HDL-C (mmol/L)	1.32 ± 0.06	1.40 ± 0.08	0.40
Dairy intake (servings/day)	0.51 ± 0.12	0.58 ± 0.13	0.34
ΔH_2_ (ppm)	73.8 ± 7.3	11.7 ± 0.8	<0.01

^1^, LM lactose malabsorbers, LA, lactose absorbers, BMI, body mass index, DBP, diastolic blood pressure, SBP, systolic blood pressure, FPG, fasting plasma glucose, FPI, fasting plasma insulin, HOMA-IR, homeostasis model assessment of insulin resistance, TG, triglycerides, TC, total cholesterol, LDL-C, low-density lipoprotein cholesterol, HDL-C, high-density lipoprotein cholesterol.

**Table 2:** Body composition and cardiometabolic biomarkers before and after whole milk supplementation in subjects with or without lactose malabsorption ^1^.

Parameters	LM (*n* = 31)		LA (*n* = 31)		*P*
	Pre	Post	Pre	Post	
Weight (kg)	62.8 ± 2.2	62.5 ± 2.2	64.5 ± 2.1	64.0 ± 2.2	0.55
BMI (kg/m^2^)	21.6 ± 0.6	21.5 ± 0.6	22.3 ± 0.6	22.2 ± 0.6	0.60
Body fat mass (kg)	13.6 ± 1.1	12.3 ± 1.1 *	13.9 ± 1.2	12.9 ± 1.1 **	0.54
Lean mass (kg)	27.4 ± 1.0	28.2 ± 1.1	28.5 ± 1.1	28.6 ± 1.1	0.25
Body fat (%)	21.5 ± 1.2	19.5 ± 1.3 *	21.4 ± 1.4	19.9 ± 1.3 **	0.56
DBP (mmHg)	75.6 ± 1.1	74.5 ± 1.2	75.7 ± 1.7	78.6 ± 1.6	0.15
SBP (mmHg)	115.9 ± 1.9	114.4 ± 1.7	116.2 ± 2.4	112.9 ± 2.0	0.47
FPG (mmol/L)	5.14 ± 0.06	5.20 ± 0.06	5.15 ± 0.07	5.28 ± 0.07	0.45
FPI (mU/L)	5.72 ± 0.63	6.22 ± 0.59	5.86 ± 0.64	5.48 ± 0.53	0.78
HOMA-IR	1.30 ± 0.15	1.45 ± 0.15	1.36 ± 0.16	1.22 ± 0.14	0.39
C-peptide (nmol/L)	0.45 ± 0.03	0.44 ± 0.03	0.45 ± 0.04	0.43 ± 0.03	0.84
TG (mmol/L)	0.74 ± 0.07	0.77 ± 0.07	0.86 ± 0.06	0.92 ± 0.09	0.85
TC (mmol/L)	4.22 ± 0.15	4.08 ± 0.14	4.19 ± 0.22	3.88 ± 0.16	0.93
LDL-C (mmol/L)	2.52 ± 0.14	2.52 ± 0.13	2.39 ± 0.14	2.50 ± 0.13	0.42
HDL-C (mmol/L)	1.32 ± 0.06	1.33 ± 0.07	1.40 ± 0.08	1.36 ± 0.07	0.52
CRP (μg/mL)	0.68 ± 0.22	0.81 ± 0.22	0.80 ± 0.31	0.66 ± 0.20	0.71
MDA (nmol/mL)	4.95 ± 0.19	4.87 ± 0.19	4.92 ± 0.19	4.84 ± 0.15	0.98

^1^, LM, lactose malabsorbers, LA, lactose absorbers, BMI, body mass index, DBP diastolic blood pressure, SBP systolic blood pressure, FPG, fasting plasma glucose, FPI, fasting plasma insulin, HOMA-IR, homeostasis model assessment of insulin resistance, TG, triglycerides, TC, total cholesterol, LDL-C, low-density lipoprotein cholesterol, HDL-C, high-density lipoprotein cholesterol, CRP, C-reactive protein, MDA, malondialdehyde. *, *P* < 0.05; **, *P* < 0.01.

## Supplementary Materials

The following are available online at http://www.mdpi.com/2072-6643/10/10/1403/s1, Figure S1: Flow chart of the participant recruitment and withdraw, Table S1: Dietary energy and nutrients intake based on a three-day food records at baseline and following 4-week supplementation of whole milk, Table S2: Comparison of gut microbiota composition at the phylum and genus level betweem lactose malabsorbers (LM) and absorbers (LA), Table S3: Comparison of changes in cardiometabolic biomarkers between groups based on changes in *Bifidobacterium* abundance among lactose malabsorbers, Table S4: Comparison of changes in cardiometabolic biomarkers between groups based on changes in *Anaerostipes* abundance among lactose malabsorbers, Table S5: Comparison of changes in cardiometabolic biomarkers between groups based on changes in *Blautia* abundance among lactose malabsorbers, Table S6: Comparison of changes in cardiometabolic biomarkers between groups based on changes in *Megamonas* abundance among lactose malaborbers.
