# Supplementary material for: Effects of Whole Milk Supplementation on Gut Microbiota and Cardiometabolic Biomarkers in Subjects with and without Lactose Malabsorption
Source: Nutrients. 2018 Oct 2;10(10):1403. doi: 10.3390/nu10101403 (PMC6213503; doi:10.3390/nu10101403)
Supplement: Supplementary file 1 [file nutrients-10-01403-s001.pdf]

Supplementary material

Supplementary figure

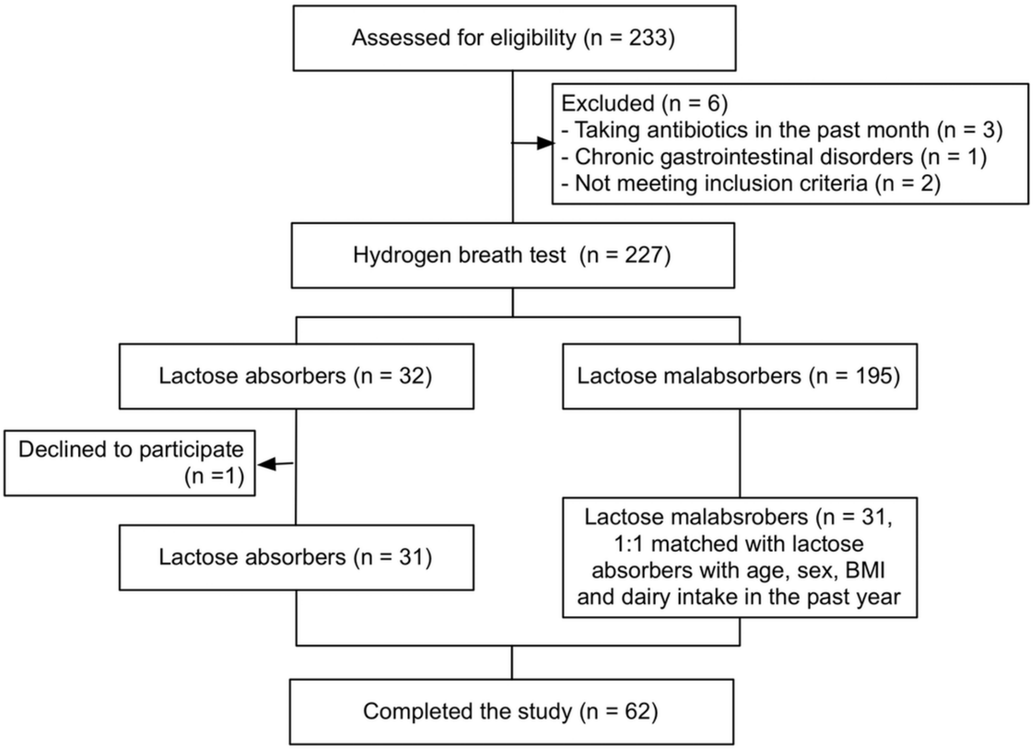

Figure S1. Flow chart of the participant recruitment and withdraw

Supplementary tables

Table S1. Dietary energy and nutrients intake based on a three-day food records at baseline and following 4-week supplementation of whole milk[1]

Nutrients	LM (*n*=31)		LA (*n*=31)	
	Pre	Post	Pre	Post
Energy (Kcal)	2035 ± 47	2089 ± 61	1981 ± 52	2046 ± 64
Protein (g)	64.5 ± 2.7	66.4 ± 2.8	63.2 ± 3.6	63.8 ± 4.3
Protein (E %)	12.7 ± 0.4	12.6 ± 0.3	12.7 ± 0.4	12.5 ± 0.5
Fat (g)	81.4 ± 2.4	86.3 ± 3.0	79.3 ± 2.9	84.3 ± 2.9
Fat (E %)	36.1 ± 0.7	37.3 ± 0.8	36.0 ± 1.0	37.1 ± 0.8
Carbohydrate (g)	261.0 ± 7.3	261.0 ± 9.2	250.6 ± 7.8	257.8 ± 8.7
Carbohydrate (E %)	51.3 ± 0.7	49.8 ± 0.9	50.7 ± 1.1	50.4 ± 1.0
Fiber (g)	8.6 ± 0.6	8.2 ± 0.7	9.4 ± 0.9	8.6 ± 0.5
Calcium (mg)	349 ± 26	568 ± 25*	344 ± 28	541 ± 29*

[1]*LM* lactose malabosrbers, *LA* lactose absorbers, *E* % energy percentage. *$P < 0.05$.

Table S2. Comparison of gut microbiota composition at the phylum and genus level betweem lactose malabsorbers (LM) and absorbers (LA)

**Taxa**	**LM (*n*=31)**	**LA (*n*=29)**	***P***
Phylum			
Firmicutes (%)	59.6 ± 2.5	61.4 ± 3.0	0.34
Bacteroidetes (%)	36.5 ± 2.5	35.1 ± 3.0	0.42
Proteobacteria (%)	2.2 ± 0.5	2.1 ± 0.5	0.91
Actinobacteria (%)	0.8 ± 0.2	0.9 ± 0.2	0.77
Genus			
Bacteroides (%)	23.0 ± 2.6	18.5 ± 2.7	0.23
Prevotella (%)	10.3 ± 3.1	14.0 ± 3.8	0.57
Faecalibacterium (%)	14.9 ± 1.8	11.0 ± 1.5	0.95
Megamonas (%)	7.8 ± 2.6	9.9 ± 2.3	0.80
Roseburia (%)	8.6 ± 1.3	9.3 ± 1.5	0.85
Clostridium (%)	2.5 ± 0.3	2.3 ± 0.3	0.15
Ruminococcus (%)	2.0 ± 0.3	3.1 ± 0.6	0.60
Dialister (%)	2.3 ± 0.6	2.5 ± 0.8	0.46
Phascolarctobacterium (%)	3.8 ± 1.2	1.0 ± 0.3	0.83
Blautia (%)	1.5 ± 0.2	2.6 ± 0.5	0.58
Eubacterium (%)	1.7 ± 0.3	2.3 ± 0.4	0.23
Gemmiger (%)	1.5 ± 0.3	1.6 ± 0.3	0.50
Bifidobacterium (%)	0.6 ± 0.2	0.5 ± 0.2	0.79
Lactobacillus (%)	0.8 ± 0.3	1.5 ± 0.7	0.61

Table S3. Comparison of changes in cardiometabolic biomarkers between groups based on changes in *Bifidobacterium* abundance among lactose malabsorbers[1]

**Parameters**	**Bif LO (*n*=16)**	**Bif HI (*n*=15)**	***P***
Weight (kg)	-0.25 ± 0.23	-0.26 ± 0.25	0.98
BMI (kg/m^2)	0.10 ± 0.16	-0.28 ± 0.13	0.07
Body fat mass (kg)	-0.59 ± 0.53	-1.99 ± 0.82	0.29
Lean mass (kg)	0.68 ± 0.65	0.85 ± 0.38	0.94
Body fat (%)	-1.11 ± 0.94	-2.95 ± 1.14	0.26
DBP (mmHg)	-3.25 ± 2.01	1.07 ± 1.78	0.12
SBP (mmHg)	-2.06 ± 2.37	-0.80 ± 2.49	0.72
FPG (mmol/L)	0.09 ± 0.08	0.03 ± 0.09	0.65
FPI (mU/L)	0.21 ± 0.79	0.81 ± 0.75	0.59
HOMA-IR	0.10 ± 0.19	0.20 ± 0.17	0.70
C-peptide (nmol/L)	-0.02 ± 0.03	0.00 ± 0.02	0.51
TG (mmol/L)	0.20 ± 0.05	0.09 ± 0.05	0.12
TC (mmol/L)	0.01 ± 0.09	-0.12 ± 0.14	0.43
LDL-C (mmol/L)	0.12 ± 0.09	-0.11 ± 0.18	0.26
HDL-C (mmol/L)	-0.03 ± 0.06	0.05 ± 0.06	0.36
CRP (μg/mL)	-0.12 ± 0.28	0.40 ± 0.37	0.43
MDA (nmol/mL)	0.24 ± 0.25	-0.42 ± 0.31	0.11

[1]*Bif LO* change in the *Bifidobacterium* abundance below the median, *Bif HI* change in the *Bifidobacterium* abundance above the median, *BMI* body mass index, *DBP* diastolic blood pressure, *SBP* systolic blood pressure, *FPG* fasting plasma glucose, *FPI* fasting plasma insulin, *HOMA-IR* homeostasis model assessment of insulin resistance, *TG* triglycerides, *TC* total cholesterol, *LDL-C* low-density lipoprotein cholesterol, *HDL-C* high-density lipoprotein cholesterol, *CRP* C-reactive protein, *MDA* malondialdehyde

Table S4. Comparison of changes in cardiometabolic biomarkers between groups based on changes in *Anaerostipes* abundance among lactose malabsorbers[1]

Parameters	Ana LO (*n*=16)	Ana HI (*n*=15)	*P*
Weight (kg)	-0.21 ± 0.23	-0.31 ± 0.25	0.77
BMI (kg/m^2)	-0.23 ± 0.12	0.07 ± 0.17	0.24
Body fat mass (kg)	-2.03 ± 0.80	-0.46 ± 0.50	0.39
Lean mass (kg)	1.38 ± 0.63	0.11 ± 0.33	0.58
Body fat (%)	-3.29 ± 1.21	-0.62 ± 0.71	0.30
DBP (mmHg)	0.50 ± 1.50	-2.93 ± 2.33	0.22
SBP (mmHg)	-0.75 ± 2.54	-2.20 ± 2.28	0.68
FPG (mmol/L)	0.07 ± 0.08	0.06 ± 0.10	0.94
FPI (mU/L)	0.87 ± 0.63	0.10 ± 0.90	0.48
HOMA-IR	0.22 ± 0.15	0.08 ± 0.21	0.59
C-peptide (nmol/L)	-0.02 ± 0.03	0.00 ± 0.03	0.54
TG (mmol/L)	0.14 ± 0.04	0.16 ± 0.06	0.82
TC (mmol/L)	-0.04 ± 0.14	-0.06 ± 0.07	0.88
LDL-C (mmol/L)	0.07 ± 0.18	-0.06 ± 0.09	0.52
HDL-C (mmol/L)	0.01 ± 0.07	0.01 ± 0.06	1.00
CRP (µg/mL)	0.45 ± 0.40	-0.20 ± 0.19	0.82
MDA (nmol/mL)	0.01 ± 0.27	-0.17 ± 0.31	0.66

[1]*Ana LO* change in the *Anaerostipes* abundance below the median, *Ana HI* change in the *Anaerostipes* abundance above the median, *BMI* body mass index, *DBP* diastolic blood pressure, *SBP* systolic blood pressure, *FPG* fasting plasma glucose, *FPI* fasting plasma insulin, *HOMA-IR* homeostasis model assessment of insulin resistance, *TG* triglycerides, *TC* total cholesterol, *LDL-C* low-density lipoprotein cholesterol, *HDL-C* high-density lipoprotein cholesterol, *CRP* C-reactive protein, *MDA* malondialdehyde

Table S5. Comparison of changes in cardiometabolic biomarkers between groups based on changes in *Blautia* abundance among lactose malabsorbers[1]

Parameters	Bla LO (*n*=16)	Bla HI (*n*=15)	*P*
Weight (kg)	-0.36 ± 0.21	-0.15 ± 0.26	0.54
BMI (kg/m^2)	-0.12 ± 0.07	-0.05 ± 0.21	0.92
Body fat mass (kg)	-0.84 ± 0.21	-1.73 ± 0.99	0.49
Lean mass (kg)	0.34 ± 0.11	1.22 ± 0.77	0.24
Body fat (%)	-1.21 ± 0.27	-2.85 ± 1.50	0.33
DBP (mmHg)	-2.88 ± 1.88	0.67 ± 1.99	0.21
SBP (mmHg)	-2.94 ± 2.58	0.13 ± 2.17	0.37
FPG (mmol/L)	0.03 ± 0.09	0.10 ± 0.07	0.58
FPI (mU/L)	0.54 ± 0.81	0.45 ± 0.73	0.93
HOMA-IR	0.16 ± 0.20	0.15 ± 0.17	0.97
C-peptide (nmol/L)	0.00 ± 0.03	-0.02 ± 0.03	0.58
TG (mmol/L)	0.19 ± 0.05	0.11 ± 0.05	0.24
TC (mmol/L)	-0.09 ± 0.08	-0.01 ± 0.14	0.59
LDL-C (mmol/L)	0.25 ± 0.09	-0.25 ± 0.16	**0.01**
HDL-C (mmol/L)	-0.04 ± 0.06	0.07 ± 0.06	0.23
CRP (μg/mL)	0.45 ± 0.42	-0.20 ± 0.15	0.32
MDA (nmol/mL)	0.06 ± 0.30	-0.23 ± 0.28	0.47

[1]*Bla LO* change in the *Blautia* abundance below the median, *Bla HI* change in the *Blautia* abundance above the median, *BMI* body mass index, *DBP* diastolic blood pressure, *SBP* systolic blood pressure, *FPG* fasting plasma glucose, *FPI* fasting plasma insulin, *HOMA-IR* homeostasis model assessment of insulin resistance, *TG* triglycerides, *TC* total cholesterol, *LDL-C* low-density lipoprotein cholesterol, *HDL-C* high-density lipoprotein cholesterol, *CRP* C-reactive protein, *MDA* malondialdehyde

Table S6. Comparison of changes in cardiometabolic biomarkers between groups based on changes in *Megamonas* abundance among lactose malaborbers[1]

Parameters	Meg LO (*n*=16)	Meg HI (*n*=15)	*P*
Weight (kg)	-0.31 ± 0.14	-0.19 ± 0.32	0.74
BMI (kg/m^2)	-0.11 ± 0.05	-0.07 ± 0.22	0.97
Body fat mass (kg)	-0.71 ± 0.19	-1.87 ± 0.99	0.15
Lean mass (kg)	0.28 ± 0.08	1.29 ± 0.76	0.07
Body fat (%)	-1.08 ± 0.25	-2.98 ± 1.49	0.11
DBP (mmHg)	0.38 ± 1.77	-2.80 ± 2.12	0.26
SBP (mmHg)	-2.13 ± 1.84	-0.73 ± 2.95	0.69
FPG (mmol/L)	0.02 ± 0.10	0.11 ± 0.06	0.43
FPI (mU/L)	0.18 ± 0.54	0.84 ± 0.97	0.55
HOMA-IR	0.06 ± 0.13	0.25 ± 0.23	0.48
C-peptide (nmol/L)	-0.02 ± 0.03	0.00 ± 0.03	0.73
TG (mmol/L)	0.12 ± 0.05	0.19 ± 0.05	0.32
TC (mmol/L)	-0.03 ± 0.06	-0.07 ± 0.15	0.80
LDL-C (mmol/L)	0.17 ± 0.10	-0.17 ± 0.16	0.09
HDL-C (mmol/L)	0.04 ± 0.07	-0.01 ± 0.05	0.60
CRP (μg/mL)	0.38 ± 0.35	-0.13 ± 0.29	0.91
MDA (nmol/mL)	-0.10 ± 0.33	-0.07 ± 0.23	0.94

[1]*Meg LO* change in the *Megamonas* abundance below the median, *Meg HI* change in the *Megamonas* abundance above the median, *BMI* body mass index, *DBP* diastolic blood pressure, *SBP* systolic blood pressure, *FPG* fasting plasma glucose, *FPI* fasting plasma insulin, *HOMA-IR* homeostasis model assessment of insulin resistance, *TG* triglycerides, *TC* total cholesterol, *LDL-C* low-density lipoprotein cholesterol, *HDL-C* high-density lipoprotein cholesterol, *CRP* C-reactive protein, *MDA* malondialdehyde
